# Crystal structure of 6,7-dimeth­oxy-1-(4-nitro­phen­yl)quinolin-4(1*H*)-one: a mol­ecular scaffold for potential tubulin polymerization inhibitors

**DOI:** 10.1107/S2056989017002948

**Published:** 2017-02-24

**Authors:** Vegard Torp Lien, Dag Erlend Olberg, Jo Klaveness, Carl Henrik Görbitz

**Affiliations:** a Department of Pharmaceutical Chemistry, University of Oslo, PO Box 1068 Blindern, N-0371 Oslo, Norway; b Department of Chemistry, University of Oslo, PO Box 1033 Blindern, N-0315 Oslo, Norway

**Keywords:** crystal structure, cytotoxic agents, *N*-substituted quinolone, tubulin polymerization, hydrogen bonding

## Abstract

The protein tubulin is central for maintaining normal cellular processes, and mol­ecules inter­fering with the tubulin dynamics have potential in the treatment of cancerous diseases. The title compound, C_17_H_14_N_2_O_5_, was prepared as a lead compound in a project dedicated to the development of therapeutic agents binding to the colchicine binding site on tubulin, thereby inter­fering with the cell division in cancer cells. It holds many of the main structural characteristics for colchicine binding and has the potential for further modification and functionalization. In the title mol­ecule, the benzene ring is inclined to the quinoline ring by 76.10 (8)°. In the crystal, mol­ecules are linked by two pairs of C—H⋯O hydrogen bonds, forming tubular-like arrangements, propagating along the direction of the diagonals of the *ab* plane, and enclosing *R*_2_^2^(26) and *R*_2_^2^(16) ring motifs.

## Abstract 2

The single-crystal X-ray diffraction investigation of a substituted quinoline derivative, which may serve as a basis for the development of a family of cytotoxic agents, confirms the anti­cipated covalent structure with an unusual twisted conformation and reveals a densely packed mol­ecular lattice.

## Chemical context   

Due to the elevated rate of cell division in cancer cells, agents targeting proteins central to the mitotic process are attractive for cancer treatment (Hanahan & Weinberg, 2011 ▸). The protein tubulin polymerizes during the mitotic phase into microtubules, and this process is vital for the correct cell division (Parker *et al.*, 2014 ▸). Based on the structures of the natural products colchicine and comberastatin A-4, a great amount of research on the synthesis and biological evaluation has been carried out (Lu *et al.*, 2012 ▸). All these analogs bind to the colchicine binding site, and the pharmacophore and binding site is well known (Nguyen *et al.*, 2005 ▸).
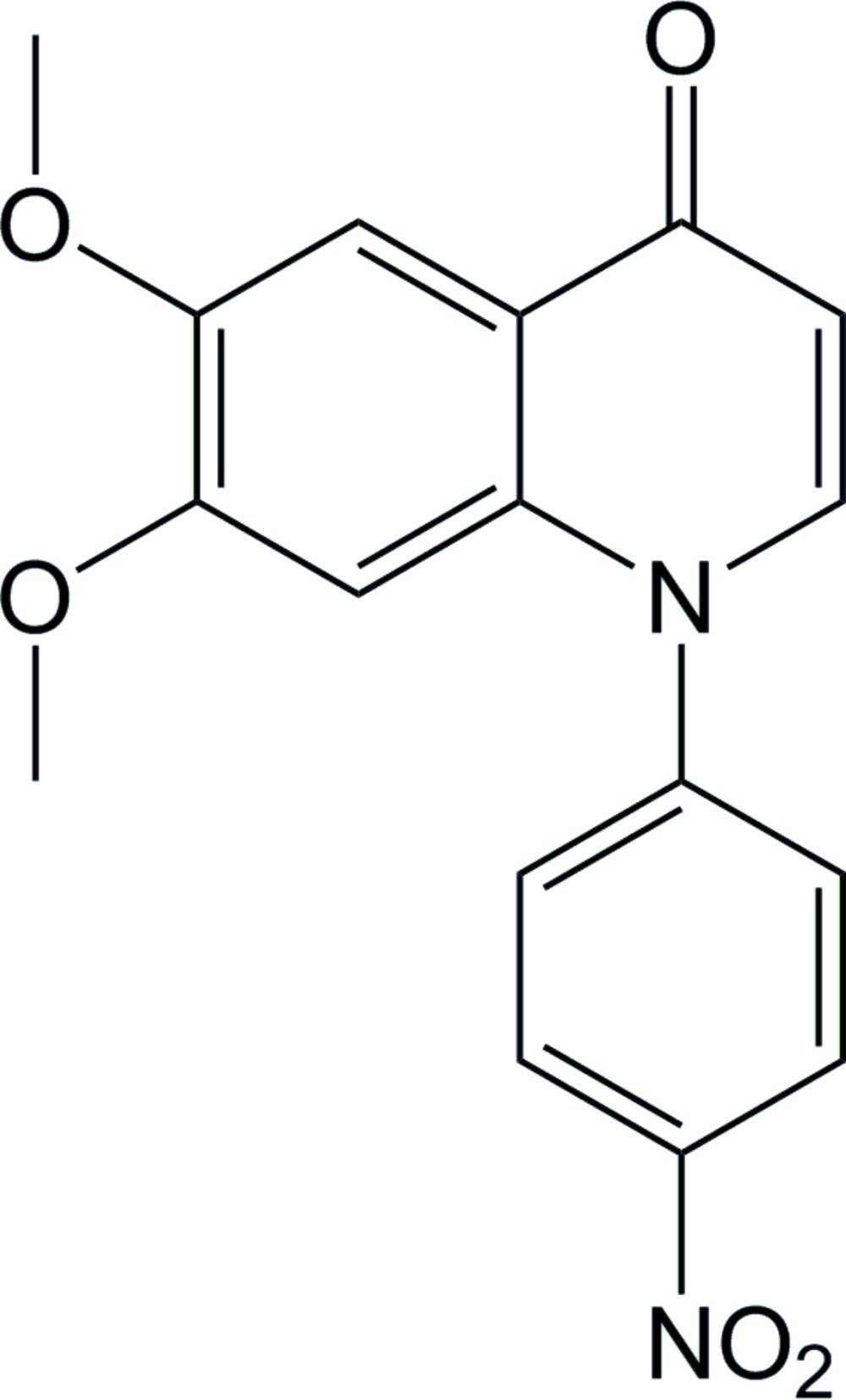


Despite large research efforts, many colchicine-binding drug candidates suffer from resistance and toxicity problems (Lu *et al.*, 2012 ▸). Therefore, further exploration and biological evaluation of possible structures is needed. From another medicinal chemistry project in our group, the title compound, (I), appeared as a side product in significant amounts. The structure was rationalized from NMR studies and confirmed by X-ray crystallography. Based on the literature and knowledge of the characteristics of mol­ecules binding to the colchicine binding site on tubulin, it is reasonable that analogs of this structure might be potent cytotoxic agents. The reported structure can easily be further modified to improve binding affinities in correspondence with reported structure–activity studies (Lai *et al.*, 2011 ▸; Wang *et al.*, 2013 ▸; Patil *et al.*, 2012 ▸). Herein, we present the synthesis and the crystal structure of the title compound, 6,7-dimeth­oxy-1-(4-nitro­phen­yl)quinolin-4(1*H*)-one (I).

## Database survey   

**Figure 1:**
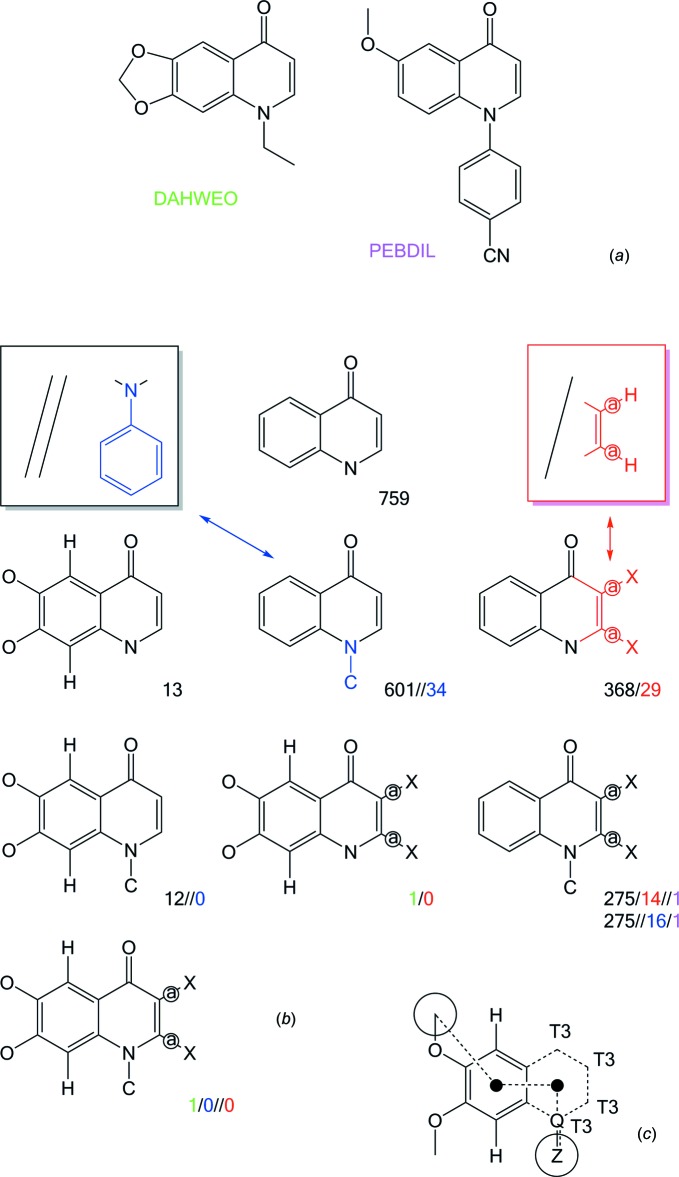
(*a*) Schematic drawing of two analogues of (I) in the Cambridge Structural Database (CSD, Version 5.37; Groom *et al.*, 2016 ▸) identified by their six-letter reference codes. (*b*) Number of entries in the CSD retrieved by using various search fragments. The raw quinolin-4(1*H*)-one skeleton (with potential substituents on all C and N atoms) yields 759 hits (including a small number of duplicates). Three types of specifications and combinations thereof are then explored: introduction of bonds to O atoms (–OH, alk­oxy or phen­oxy) from C6 and C7, N1-substitution (blue, subset aromatic ring), and including only acyclic bonds from C2 and C3 atoms (red, *X* = any atom type, subset H only). Green and violet colours indicate the two mol­ecules in (*a*). (*c*) Final CSD search fragment used in the conformational analysis. Dashed bonds have bond type ‘any’, Q is N or C, *Z* is ‘not hydrogen’, while T3 means the atom has three bonded atoms. The indicated torsion angle runs between the encircled atoms through the two ring centroids.

The frequencies of mol­ecules in the Cambridge Structural Database (CSD, version 5.37; Groom *et al.*, 2016 ▸) incorporating various modifications of the quinolin-4(1*H*)-one fragment are shown in Fig. 1 ▸ *b*. It can be seen that only one previous compound, 4-[6-meth­oxy-4-oxoquinolin-1(4*H*)-yl]benzo­nitrile (CSD refcode PEBDIL; Hirano *et al.*, 2008 ▸) share with (I) the lack of substituents at C2 and C3 as well as having an aromatic N-substituent, while 1-ethyl-1,4-di­hydro-6,7-methyl­enedi­oxy-4-oxo-3-quinoline­carb­oxy­lic acid (CSD refcode DAHWEO; Cygler & Huber, 1985 ▸) is alone in incorporating C2—H, C3—H, C6—O and C7—O bonds (Fig. 1 ▸ *a*). Even though (I) is a rather simple covalent structure, it thus represents a rather unique combination of functionalities.

## Structural commentary   

**Figure 2:**
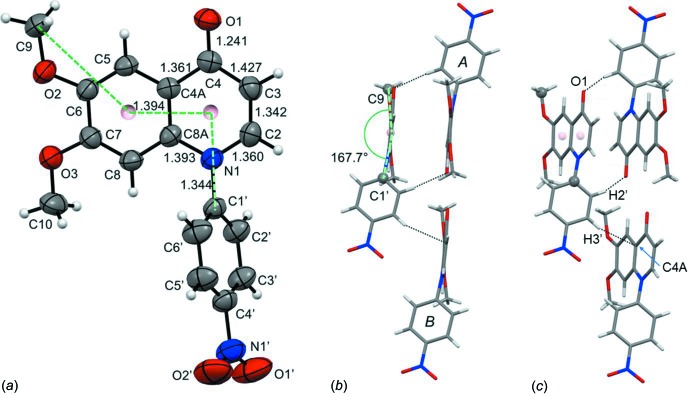
(*a*) The mol­ecular structure of (I) with some selected bond lengths (Å; s.u.’s = 0.002 Å) at 295 K. Displacement ellipsoids are shown at the 50% probability level. Pink spheres are the centroids for the two six-membered rings, and the dashed green lines defines the torsion angle discussed in the text. (*b*) View along the centroid–centroid vector showing the torsion angle from (*a*) and two neighbouring mol­ecules *A* and *B* at (−*x* + 1, −*y* + 2, −*z* + 1) and (*x* − 1, *y*, *z*), respectively. (*c*) As in (*b*), but rotated *ca* 27° around the vertical axis to display two short inter­molecular inter­actions involving the nitro­phenyl substituent; H2′⋯O1(−*x* + 1, −*y* + 2, −*z* + 1) is 2.53 Å, while H3′⋯C4*A*(*x* − 1, *y*, *z*) is 2.72 Å.

The mol­ecular structure of (I) is depicted in Fig. 2 ▸ *a*, where the short, double-bond nature of the C2=C3 bond [1.342 (2) Å] is clearly visible. While the bicyclic ring systems of DAHWOE and PEBDIL (Fig. 1 ▸ *a*) are perfectly coplanar with the C6 and C7 substituents as well as the C1′-atom attached to N1, this is not the case for (I); the nitro­benzene ring is inclined to the quinoline ring system by 76.10 (8)°, and the torsion angle defined by atom C9, the two ring centroids and atom C1′ is *ca* 167.7°; see Fig. 2 ▸ *a* and 2*b*. The more extended search fragment in Fig. 1 ▸ *c* found 157 such torsion angles in 62 CSD entries, and in only nine compounds does this torsion angle deviate by more than *ca* 13.3° from planarity.

## Supra­molecular features   

**Figure 3:**
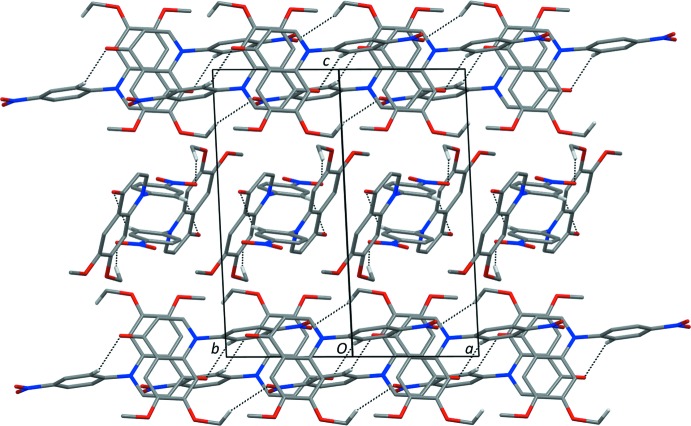
A viewed along the normal to (110) of the crystal packing of compound (I). Hydrogen bonds are shown as dashed lines (see Table 1 ▸). For clarity, only H atoms, H2′ and H103, have been included.

The reason for the unusual mol­ecular conformation of (I) can be seen in Fig. 2 ▸ *b* and 2*c*, where close contacts to two neighbouring mol­ecules are apparent; these force the meth­oxy group and the nitro­phenyl group out of the quinolinone mean plane. In the crystal, mol­ecules are linked by two pairs of C—H⋯O hydrogen bonds, forming tubular-like arrangements propagating along the direction of the diagonals of the *ab* plane, and enclosing 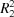
(26) and 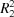
(16) ring motifs (Table 1 ▸ and Fig. 3 ▸). Within the tubular-like arrangements, mol­ecules are also linked by offset π–π inter­actions; the shortest inter­action involves inversion-related pyridine rings with an inter-centroid distance *Cg*1⋯*Cg*1(−*x* + 1, −*y* + 2, −*z* + 1) = 3.659 (1) Å [*Cg*1 is the centroid of the N1/C2–C4/C4*A*/C8*A* ring; inter­planar distance = 3.580 (1) Å, slippage = 0.754 Å]. The crystal density is comparatively high at 1.415 g cm^−3^, and no voids were calculated by *Mercury* (Macrae *et al.*, 2008 ▸) using the default settings (probe radius 1.2 Å, grid spacing 0.7 Å).

## Synthesis and crystallization   

Cs_2_CO_3_ (0.212 g, 0.65 mmol) and 6,7-di­meth­oxy­quinolin-4-ol (67 mg, 0.326 mmol) were weighed out in a round-bottom flask, to which was added 3 ml DMF and 1 ml MeCN. The mixture was then stirred for 15 min. 1-Fluoro-4-nitro­benzene (101 mg, 0.716 mmol) in 2 ml 1:1 DMF:MeCN was then added, and the reaction mixture was stirred for 20 h at 328 K. The crude product was washed with water (4 × 10 ml) and brine (10 ml), and then purified by column chromatography [Hep:EtOAc (4:1) → Hep:EtOAc:MeOH (10:10:1)]. The title compound (I) was obtained as a yellow solid (40 mg, 38%). ^1^H NMR (CDCl_3_, 400 MHz): δ 8.48 (*d*, 2H, *J* = 8.8 Hz), 7.79 (*s*, 1H), 7.67 (*d*, 2H, *J* = 8.8 Hz), 7.48 (*d*, 1H, *J* = 7.8 Hz), 6.35 (*d*, 1H, *J* = 7.7 Hz), 6.32 (*s*, 1H), 3.98 (*s*, 3H), 3.72 (*s*, 3H). ^13^C NMR (CDCl_3_, 101 MHz): δ 176.98, 153.56, 147.96, 147.71, 146.91, 140.54, 136.08, 128.64, 125.92, 120.99, 110.68, 106.17, 98.10, 56.46, 56.21. HRMS (ESI^+^) *m*/*z* calculated for C_17_H_15_N_2_O_5_ [*M*+H]^+^: 327.0975, found 327.0976. Yellow crystals of compound (I) were grown from a hepta­ne:EtOAc:MeOH (10:10:1) solution by slow evaporation of the solvent.

## 1 Refinement   

Crystal data, data collection and structure refinement details are summarized in Table 2 ▸. The H atoms were included in calculated positions and treated as riding: C—H = 0.93–0.96 Å with *U*_iso_(H) = 1.5*U*_eq_(C-meth­yl) and 1.2*U*_eq_(C) for other H atoms.

## Supplementary Material

Crystal structure: contains datablock(s) I, global. DOI: 10.1107/S2056989017002948/su5354sup1.cif

Structure factors: contains datablock(s) I. DOI: 10.1107/S2056989017002948/su5354Isup2.hkl

Supporting information file. DOI: 10.1107/S2056989017002948/su5354Isup3.cml

CCDC reference: 1533984

Additional supporting information:  crystallographic information; 3D view; checkCIF report

## Figures and Tables

**Table 1:** Hydrogen-bond geometry (Å, °)

*D*—H⋯*A*	*D*—H	H⋯*A*	*D*⋯*A*	*D*—H⋯*A*
C2′—H2′⋯O1^i^	0.93	2.53	3.320 (2)	143
C10—H103⋯O1′^ii^	0.96	2.60	3.512 (3)	160

Symmetry codes: (i) 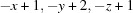; (ii) 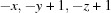.

**Table 2:** Experimental details

Crystal data	
Chemical formula	C_17_H_14_N_2_O_5_
*M*_r_	326.30
Crystal system, space group	Monoclinic, *P*2_1_/*n*
Temperature (K)	295
*a*, *b*, *c* (Å)	8.3736 (4), 11.7694 (5), 15.5623 (8)
β (°)	93.251 (1)
*V* (Å^3^)	1531.23 (13)
*Z*	4
Radiation type	Mo *K*α
μ (mm^−1^)	0.11
Crystal size (mm)	0.66 × 0.27 × 0.08

Data collection	
Diffractometer	Bruker D8 Venture diffractometer with a Photon 100 CMOS detector
Absorption correction	Multi-scan (*SADABS*; Bruker, 2016 ▸)
*T*_min_, *T*_max_	0.930, 1.000
No. of measured, independent and observed [*I* > 2σ(*I*)] reflections	20516, 3142, 2298
*R*_int_	0.032
(sin θ/λ)_max_ (Å^−1^)	0.626

Refinement	
*R*[*F* ^2^ > 2σ(*F* ^2^)], *wR*(*F* ^2^), *S*	0.047, 0.127, 1.03
No. of reflections	3142
No. of parameters	219
H-atom treatment	H-atom parameters constrained
Δρ_max_, Δρ_min_ (e Å^−3^)	0.20, −0.21

Computer programs: *APEX3* and *SAINT-Plus* (Bruker, 2016 ▸), *SHELXT2014* (Sheldrick, 2015*a* ▸), *SHELXL2014* (Sheldrick, 2015*b* ▸) and *Mercury* (Macrae *et al.*, 2008 ▸).

## supplementary crystallographic information

### Crystal data

**Table:**

C_17_H_14_N_2_O_5_	*F*(000) = 680
*M**_r_* = 326.30	*D*_x_ = 1.415 Mg m^−^^3^
Monoclinic, *P*2_1_/*n*	Mo *K*α radiation, λ = 0.71073 Å
*a* = 8.3736 (4) Å	Cell parameters from 8925 reflections
*b* = 11.7694 (5) Å	θ = 2.6–26.4°
*c* = 15.5623 (8) Å	µ = 0.11 mm^−^^1^
β = 93.251 (1)°	*T* = 295 K
*V* = 1531.23 (13) Å^3^	Flat lens, yellow
*Z* = 4	0.66 × 0.27 × 0.08 mm

### Data collection

**Table:**

Bruker D8 Venture diffractometer with a Photon 100 CMOS detector	3142 independent reflections
Radiation source: fine-focus sealed tube	2298 reflections with *I* > 2σ(*I*)
Graphite monochromator	*R*_int_ = 0.032
Detector resolution: 8.3 pixels mm^-1^	θ_max_ = 26.4°, θ_min_ = 2.2°
Sets of exposures each taken over 0.5° ω rotation scans	*h* = −10→10
Absorption correction: multi-scan (SADABS; Bruker, 2016)	*k* = −14→14
*T*_min_ = 0.930, *T*_max_ = 1.000	*l* = −19→19
20516 measured reflections	

### Refinement

**Table:**

Refinement on *F*^2^	Primary atom site location: structure-invariant direct methods
Least-squares matrix: full	Secondary atom site location: difference Fourier map
*R*[*F*^2^ > 2σ(*F*^2^)] = 0.047	Hydrogen site location: inferred from neighbouring sites
*wR*(*F*^2^) = 0.127	H-atom parameters constrained
*S* = 1.03	*w* = 1/[σ^2^(*F*_o_^2^) + (0.0557*P*)^2^ + 0.4805*P*] where *P* = (*F*_o_^2^ + 2*F*_c_^2^)/3
3142 reflections	(Δ/σ)_max_ < 0.001
219 parameters	Δρ_max_ = 0.20 e Å^−^^3^
0 restraints	Δρ_min_ = −0.21 e Å^−^^3^

### Special details

**Table:**

Geometry. All esds (except the esd in the dihedral angle between two l.s. planes) are estimated using the full covariance matrix. The cell esds are taken into account individually in the estimation of esds in distances, angles and torsion angles; correlations between esds in cell parameters are only used when they are defined by crystal symmetry. An approximate (isotropic) treatment of cell esds is used for estimating esds involving l.s. planes.

### Fractional atomic coordinates and isotropic or equivalent isotropic displacement parameters (Å^2^)

**Table:**

	*x*	*y*	*z*	*U*_iso_*/*U*_eq_	
O1	0.82921 (15)	0.99910 (12)	0.42505 (8)	0.0623 (4)	
O2	0.86545 (15)	0.77881 (14)	0.70970 (8)	0.0684 (4)	
O3	0.59678 (16)	0.67943 (13)	0.71853 (8)	0.0679 (4)	
N1	0.41894 (16)	0.81504 (12)	0.43553 (9)	0.0477 (4)	
C2	0.4438 (2)	0.88507 (16)	0.36804 (11)	0.0528 (4)	
H2	0.3660	0.8887	0.3230	0.063*	
C3	0.5757 (2)	0.94908 (16)	0.36357 (12)	0.0538 (5)	
H3	0.5850	0.9969	0.3165	0.065*	
C4	0.7020 (2)	0.94582 (15)	0.42906 (11)	0.0466 (4)	
C5	0.7864 (2)	0.86419 (15)	0.57179 (11)	0.0461 (4)	
H5	0.8813	0.9051	0.5704	0.055*	
C6	0.7612 (2)	0.79688 (16)	0.64091 (11)	0.0501 (4)	
C7	0.6135 (2)	0.73853 (16)	0.64500 (11)	0.0508 (4)	
C8	0.5013 (2)	0.74416 (16)	0.57787 (11)	0.0491 (4)	
H8	0.4056	0.7045	0.5803	0.059*	
C9	1.0221 (2)	0.8218 (2)	0.70451 (15)	0.0789 (7)	
H91	1.0657	0.7957	0.6523	0.118*	
H92	1.0879	0.7955	0.7530	0.118*	
H93	1.0191	0.9033	0.7047	0.118*	
C10	0.4431 (3)	0.6342 (2)	0.73209 (14)	0.0791 (7)	
H101	0.3652	0.6941	0.7281	0.119*	
H102	0.4437	0.6002	0.7882	0.119*	
H103	0.4164	0.5777	0.6892	0.119*	
C4A	0.67157 (18)	0.87302 (14)	0.50243 (10)	0.0422 (4)	
C8A	0.53089 (18)	0.81007 (14)	0.50514 (10)	0.0430 (4)	
O1'	−0.2498 (2)	0.57251 (18)	0.39917 (15)	0.1105 (7)	
O2'	−0.0978 (2)	0.42948 (16)	0.37958 (14)	0.1038 (6)	
N1'	−0.1179 (2)	0.52979 (17)	0.39326 (12)	0.0718 (5)	
C1'	0.2824 (2)	0.74056 (15)	0.42899 (11)	0.0460 (4)	
C2'	0.1327 (2)	0.78390 (16)	0.43907 (12)	0.0547 (5)	
H2'	0.1198	0.8597	0.4540	0.066*	
C3'	0.0017 (2)	0.71397 (17)	0.42679 (13)	0.0588 (5)	
H3'	−0.1009	0.7421	0.4327	0.071*	
C4'	0.0242 (2)	0.60273 (16)	0.40578 (12)	0.0532 (5)	
C5'	0.1729 (2)	0.55765 (18)	0.39643 (14)	0.0663 (6)	
H5'	0.1852	0.4815	0.3823	0.080*	
C6'	0.3036 (2)	0.62773 (17)	0.40849 (14)	0.0635 (5)	
H6'	0.4060	0.5992	0.4028	0.076*	

### Atomic displacement parameters (Å^2^)

**Table:**

	*U*^11^	*U*^22^	*U*^33^	*U*^12^	*U*^13^	*U*^23^
O1	0.0581 (8)	0.0703 (9)	0.0589 (8)	−0.0198 (7)	0.0065 (6)	0.0060 (7)
O2	0.0502 (7)	0.0978 (11)	0.0556 (8)	−0.0166 (7)	−0.0109 (6)	0.0185 (7)
O3	0.0605 (8)	0.0899 (10)	0.0524 (8)	−0.0207 (7)	−0.0041 (6)	0.0208 (7)
N1	0.0440 (8)	0.0555 (9)	0.0431 (8)	−0.0060 (7)	−0.0013 (6)	−0.0012 (7)
C2	0.0568 (10)	0.0594 (11)	0.0413 (10)	−0.0010 (9)	−0.0040 (8)	0.0006 (9)
C3	0.0608 (11)	0.0567 (11)	0.0439 (10)	−0.0062 (9)	0.0039 (8)	0.0050 (8)
C4	0.0493 (10)	0.0459 (9)	0.0452 (10)	−0.0034 (8)	0.0088 (7)	−0.0055 (8)
C5	0.0388 (8)	0.0533 (10)	0.0463 (10)	−0.0070 (7)	0.0041 (7)	−0.0036 (8)
C6	0.0437 (9)	0.0621 (11)	0.0438 (10)	−0.0031 (8)	−0.0022 (7)	0.0008 (8)
C7	0.0498 (10)	0.0593 (11)	0.0433 (10)	−0.0067 (8)	0.0043 (8)	0.0055 (8)
C8	0.0418 (9)	0.0583 (10)	0.0470 (10)	−0.0109 (8)	0.0023 (7)	0.0011 (8)
C9	0.0613 (13)	0.0953 (17)	0.0772 (15)	−0.0268 (12)	−0.0206 (11)	0.0183 (13)
C10	0.0775 (14)	0.1009 (18)	0.0584 (13)	−0.0415 (13)	0.0006 (10)	0.0181 (12)
C4A	0.0407 (8)	0.0448 (9)	0.0414 (9)	−0.0013 (7)	0.0061 (7)	−0.0053 (7)
C8A	0.0403 (9)	0.0494 (10)	0.0391 (9)	0.0002 (7)	0.0021 (7)	−0.0051 (7)
O1'	0.0539 (10)	0.1104 (14)	0.165 (2)	−0.0167 (10)	−0.0103 (10)	−0.0218 (13)
O2'	0.0966 (13)	0.0717 (12)	0.1419 (18)	−0.0284 (10)	−0.0051 (11)	−0.0223 (11)
N1'	0.0650 (12)	0.0740 (13)	0.0750 (12)	−0.0178 (10)	−0.0073 (9)	−0.0091 (10)
C1'	0.0446 (9)	0.0528 (10)	0.0402 (9)	−0.0043 (8)	−0.0018 (7)	−0.0047 (8)
C2'	0.0497 (10)	0.0523 (10)	0.0616 (12)	0.0017 (8)	−0.0004 (8)	−0.0110 (9)
C3'	0.0430 (10)	0.0652 (12)	0.0681 (13)	0.0017 (9)	0.0013 (9)	−0.0098 (10)
C4'	0.0504 (10)	0.0596 (11)	0.0489 (10)	−0.0094 (9)	−0.0037 (8)	−0.0077 (9)
C5'	0.0638 (12)	0.0512 (11)	0.0842 (15)	−0.0031 (10)	0.0050 (10)	−0.0163 (10)
C6'	0.0481 (10)	0.0614 (12)	0.0813 (14)	0.0039 (9)	0.0061 (9)	−0.0145 (11)

### Geometric parameters (Å, º)

**Table:**

O1—C4	1.241 (2)	C9—H91	0.9600
O2—C6	1.359 (2)	C9—H92	0.9600
O2—C9	1.412 (2)	C9—H93	0.9600
O3—C7	1.353 (2)	C10—H101	0.9600
O3—C10	1.419 (2)	C10—H102	0.9600
N1—C2	1.360 (2)	C10—H103	0.9600
N1—C8A	1.393 (2)	C4A—C8A	1.394 (2)
N1—C1'	1.440 (2)	O1'—N1'	1.222 (2)
C2—C3	1.342 (2)	O2'—N1'	1.213 (2)
C2—H2	0.9300	N1'—C4'	1.471 (2)
C3—C4	1.427 (3)	C1'—C2'	1.370 (2)
C3—H3	0.9300	C1'—C6'	1.380 (3)
C4—C4A	1.461 (2)	C2'—C3'	1.376 (3)
C5—C6	1.362 (2)	C2'—H2'	0.9300
C5—C4A	1.408 (2)	C3'—C4'	1.365 (3)
C5—H5	0.9300	C3'—H3'	0.9300
C6—C7	1.419 (2)	C4'—C5'	1.369 (3)
C7—C8	1.366 (2)	C5'—C6'	1.375 (3)
C8—C8A	1.406 (2)	C5'—H5'	0.9300
C8—H8	0.9300	C6'—H6'	0.9300
			
C6—O2—C9	117.12 (15)	O3—C10—H102	109.5
C7—O3—C10	117.17 (15)	H101—C10—H102	109.5
C2—N1—C8A	120.01 (14)	O3—C10—H103	109.5
C2—N1—C1'	118.03 (14)	H101—C10—H103	109.5
C8A—N1—C1'	121.74 (14)	H102—C10—H103	109.5
C3—C2—N1	122.86 (16)	C8A—C4A—C5	118.63 (15)
C3—C2—H2	118.6	C8A—C4A—C4	121.33 (15)
N1—C2—H2	118.6	C5—C4A—C4	120.03 (15)
C2—C3—C4	121.77 (17)	N1—C8A—C4A	119.12 (15)
C2—C3—H3	119.1	N1—C8A—C8	120.52 (15)
C4—C3—H3	119.1	C4A—C8A—C8	120.35 (15)
O1—C4—C3	123.65 (16)	O2'—N1'—O1'	123.3 (2)
O1—C4—C4A	121.58 (16)	O2'—N1'—C4'	118.15 (19)
C3—C4—C4A	114.77 (15)	O1'—N1'—C4'	118.53 (19)
C6—C5—C4A	121.24 (15)	C2'—C1'—C6'	121.02 (17)
C6—C5—H5	119.4	C2'—C1'—N1	119.55 (16)
C4A—C5—H5	119.4	C6'—C1'—N1	119.35 (16)
O2—C6—C5	126.29 (16)	C1'—C2'—C3'	119.25 (17)
O2—C6—C7	114.29 (15)	C1'—C2'—H2'	120.4
C5—C6—C7	119.43 (16)	C3'—C2'—H2'	120.4
O3—C7—C8	124.88 (16)	C4'—C3'—C2'	119.14 (17)
O3—C7—C6	114.76 (15)	C4'—C3'—H3'	120.4
C8—C7—C6	120.36 (16)	C2'—C3'—H3'	120.4
C7—C8—C8A	119.83 (16)	C3'—C4'—C5'	122.41 (17)
C7—C8—H8	120.1	C3'—C4'—N1'	118.02 (17)
C8A—C8—H8	120.1	C5'—C4'—N1'	119.57 (18)
O2—C9—H91	109.5	C4'—C5'—C6'	118.35 (18)
O2—C9—H92	109.5	C4'—C5'—H5'	120.8
H91—C9—H92	109.5	C6'—C5'—H5'	120.8
O2—C9—H93	109.5	C5'—C6'—C1'	119.82 (18)
H91—C9—H93	109.5	C5'—C6'—H6'	120.1
H92—C9—H93	109.5	C1'—C6'—H6'	120.1
O3—C10—H101	109.5		
			
C8A—N1—C2—C3	1.9 (3)	C1'—N1—C8A—C8	−9.9 (2)
C1'—N1—C2—C3	−172.90 (17)	C5—C4A—C8A—N1	−177.76 (15)
N1—C2—C3—C4	1.6 (3)	C4—C4A—C8A—N1	1.4 (2)
C2—C3—C4—O1	176.06 (18)	C5—C4A—C8A—C8	3.4 (2)
C2—C3—C4—C4A	−3.3 (3)	C4—C4A—C8A—C8	−177.44 (15)
C9—O2—C6—C5	−8.6 (3)	C7—C8—C8A—N1	178.84 (16)
C9—O2—C6—C7	171.73 (18)	C7—C8—C8A—C4A	−2.3 (3)
C4A—C5—C6—O2	177.51 (17)	C2—N1—C1'—C2'	−75.8 (2)
C4A—C5—C6—C7	−2.9 (3)	C8A—N1—C1'—C2'	109.47 (19)
C10—O3—C7—C8	−9.8 (3)	C2—N1—C1'—C6'	101.0 (2)
C10—O3—C7—C6	170.42 (18)	C8A—N1—C1'—C6'	−73.7 (2)
O2—C6—C7—O3	3.4 (2)	C6'—C1'—C2'—C3'	−1.3 (3)
C5—C6—C7—O3	−176.25 (17)	N1—C1'—C2'—C3'	175.48 (17)
O2—C6—C7—C8	−176.36 (18)	C1'—C2'—C3'—C4'	0.7 (3)
C5—C6—C7—C8	4.0 (3)	C2'—C3'—C4'—C5'	0.1 (3)
O3—C7—C8—C8A	178.89 (17)	C2'—C3'—C4'—N1'	179.83 (18)
C6—C7—C8—C8A	−1.4 (3)	O2'—N1'—C4'—C3'	−175.7 (2)
C6—C5—C4A—C8A	−0.8 (2)	O1'—N1'—C4'—C3'	2.5 (3)
C6—C5—C4A—C4	−179.93 (16)	O2'—N1'—C4'—C5'	4.1 (3)
O1—C4—C4A—C8A	−177.59 (16)	O1'—N1'—C4'—C5'	−177.7 (2)
C3—C4—C4A—C8A	1.8 (2)	C3'—C4'—C5'—C6'	−0.2 (3)
O1—C4—C4A—C5	1.6 (2)	N1'—C4'—C5'—C6'	179.99 (19)
C3—C4—C4A—C5	−179.05 (16)	C4'—C5'—C6'—C1'	−0.4 (3)
C2—N1—C8A—C4A	−3.3 (2)	C2'—C1'—C6'—C5'	1.1 (3)
C1'—N1—C8A—C4A	171.27 (15)	N1—C1'—C6'—C5'	−175.66 (18)
C2—N1—C8A—C8	175.52 (16)		

### Hydrogen-bond geometry (Å, º)

**Table:**

*D*—H···*A*	*D*—H	H···*A*	*D*···*A*	*D*—H···*A*
C2′—H2′···O1^i^	0.93	2.53	3.320 (2)	143
C10—H103···O1′^ii^	0.96	2.60	3.512 (3)	160

Symmetry codes: (i) −*x*+1, −*y*+2, −*z*+1; (ii) −*x*, −*y*+1, −*z*+1.
